# Flavonoids and Ellagitannins Characterization, Antioxidant and Cytotoxic Activities of *Phyllanthus acuminatus* Vahl

**DOI:** 10.3390/plants6040062

**Published:** 2017-12-15

**Authors:** Mirtha Navarro, Ileana Moreira, Elizabeth Arnaez, Silvia Quesada, Gabriela Azofeifa, Felipe Vargas, Diego Alvarado, Pei Chen

**Affiliations:** 1 Department of Chemistry, University of Costa Rica (UCR), Rodrigo Facio Campus, San Pedro Montes Oca, San Jose 2060, Costa Rica; luis.vargashuertas@ucr.ac.cr; 2 Department of Biology, Technological University of Costa Rica (TEC), Cartago 7050, Costa Rica; imoreira@itcr.ac.cr (I.M.); earnaez@itcr.ac.cr (E.A.); 3 Department of Biochemistry, School of Medicine, University of Costa Rica (UCR), Rodrigo Facio Campus, San Pedro Montes Oca, San Jose 2060, Costa Rica; silvia.quesada@ucr.ac.cr (S.Q.); gabriela.azofeifacordero@ucr.ac.cr (G.A.); 4 Department of Biology, University of Costa Rica (UCR), Rodrigo Facio Campus, San Pedro Montes Oca, San Jose 2060, Costa Rica; luis.alvaradocorella@ucr.ac.cr; 5 Food Composition and Methods Development Laboratory, Department of Agriculture, Beltsville Human Nutrition Research Center, Agricultural Research Service, Beltsville, MD 20705, USA; pei.chen@ars.usda.gov

**Keywords:** *P. acuminatus*, UPLC, ESI-MS, flavonoids, ellagitannins, mass spectrometry, antioxidant, cytotoxicity

## Abstract

The phenolic composition of leaves from *Phyllanthus acuminatus* L., a plant commonly used in Costa Rica as traditional medicine, was studied using UPLC-ESI-MS on an enriched phenolic extract. A total of 20 phenolic compounds were identified, comprising eight flavonoids (two flavanones—pinocembrin isomers and six derivatives from apigenin, chrysin, quercetin, and kaempferol); seven ellagitannins, two flavan-3-ols (prodelphinidin B dimer and (epi)gallocatechin); and three phenolic acids (ellagic acid, trimethylellagic acid, and ferulic acid). All of these compounds are reported for the first time in *P. acuminatus*, while previously reported in the genus *Phyllanthus*. Antioxidant evaluation was performed for *P. acuminatus* phenolic extract obtaining DPPH results with a remarkably low IC_50_ value of 0.15 μg/mL. Also, cytotoxicity on gastric AGS and colon SW20 adenocarcinoma cell lines was evaluated, and highly promising results were obtained, with IC_50_ values of 11.3 μg/mL and 10.5 μg/mL, respectively. Furthermore, selectivity index values obtained when comparing cytotoxicity on normal Vero cells was SI > 20 for both cancer cell lines, indicating a particularly high selectivity. Additionally, Justicidin B, a metabolite extensively studied for its antitumoral activity, was isolated from a non-polar extract of *P. acuminatus*, and comparatively evaluated for both bioactivities. The DPPH value obtained for Justicidin B was moderate (IC_50_ = 14.28 μg/mL), while cytotoxicity values for both AGS (IC_50_ = 19.5 μg/mL) and SW620 (IC_50_ = 24.8 μg/mL) cell lines, as well as selectivity when compared with normal Vero cells (SI = 5.4 and 4.2 respectively), was good, but lower than *P. acuminatus* extract. These preliminary results suggest that *P. acuminatus* enriched phenolic extract containing flavonoids, ellagitannins, flavan-3-ols, and phenolic acids, reported for the first time in this plant, could be of interest for further cancer cytotoxicity studies to elucidate structure–bioactivity relationships, and the molecular mechanisms and pathways.

## 1. Introduction

The search for new drugs, or the combinatory effects of these, looking for a synergistic effect that can target several human health problems, is urged and mandatory. Efforts on synthetic medication to treat cancer and tumorigenesis, in recent years, have involved mainly the approach of changing functional groups on natural precursors in order to augment the potency of their effects. However, despite relative efficiency in treating targeted diseases, their use remains associated with important collateral effects.

Extracts of several botanical origins have been shown as promising resources for obtaining new isolated metabolites, as well as sources of mixtures of compounds with differential and synergistic effects at biochemical, cellular, and physiological levels. Synergic assessment of plant polyphenols, particularly flavonoids, has taught us that often centuries-old multi-drug combinations of traditional medicine are superior to the single modified constituent trends observed in recent literature and medical practice [1].

*Phyllanthus acuminatus* belongs to the most diversified genus of Phyllantaceae family *sensu lato*, which is widespread globally, and comprises circa 14 different species in Costa Rica [2], however, this plant has been particularly used in traditional medicine at local level [3]. *P. acuminatus* is used as icthioside piscicide, in order to hunt fishes [4], suggesting an important insight on its biological activity. In fact, both, antitumor and antimalarial activities have been reported [5]. Regarding metabolites, a majority of lignans has been reported in this plant, such as phyllanthocindiols, deacetylphyllanthostatins, and deacetylphyllanthosides [6], as well as phyllanthostatins and phyllantosides, including Justicidin B [7,8,9], with attributed potent antitumoral activity.

In recent literature, sufficient evidence on polyphenols has been reported to be considered a serious option for the management of non-communicable diseases, such as cancer and infections, supporting the potential use of complex multi-target approach to treat diseases with polyphenols. In fact, there is increasing evidence that these compounds have multiple molecular targets, modulate pro-inflammatory gene expression, also interacting with phospholipid membranes [10], and modulating pathways related to chronic inflammation and energy metabolism [11], among their studied anticancer activity [12].

The literature reported for polyphenols in *P. acuminatus* is scarce, as well as their antioxidant and cytotoxic effects. Most studies regarding antineoplastic activity in vivo and in vitro have been performed with the above-mentioned lignan metabolites. For instance, since the first study in murine lymphocyte leukemia P388 [7], among aryl naphthalene lignans, Justicidin B has been of special interest, because of its promising effects in cancer cells. For instance, this lignan showed strong cytotoxic effects on chronic myeloid leukemia (LAMA-8 and K-562) and chronic lymphoid leukemia (SKW-3) [13], and furthermore, it induced programmed cell death on breast cancer cell lines MDA-MB-231 and MCF-7 [14], and exhibited antiplatelet potency [15].

There are no specific reports on the ecological interaction of flavonoids from *P. acuminatus* and from *Phyllanthus* genus. However, flavonoids have been reported to have a role protecting plants against insect pests, influencing their behavior, growth, and development [16], and against UV damage, acting as screens that absorb UV radiation and transfer light energy to or from other molecules, via sensitization [17]; and to act as antipathogenic compounds that can be non-specific and result from their antioxidant properties [18].

This evidence and the scarcity of phenolic data lead us to perform a comparative study, aiming to obtain an enriched phenolic extract of *P. acuminatus*, in order to characterize its phenolic contents through UPLC-ESI-MS, and to evaluate its antioxidant properties and cytotoxic activity on gastric AGS and colon SW620 cancer cell lines, specially targeted by polyphenols [19]; as well as to isolate Justicidin B, reported as a highly potent antitumoral metabolite, in order to assess also its bioactivity effects, and to perform comparative data analysis.

## 2. Results and Discussion

### 2.1. Phenolic Profile by UPLC-ESI-MS Analysis

**Figure 1:**
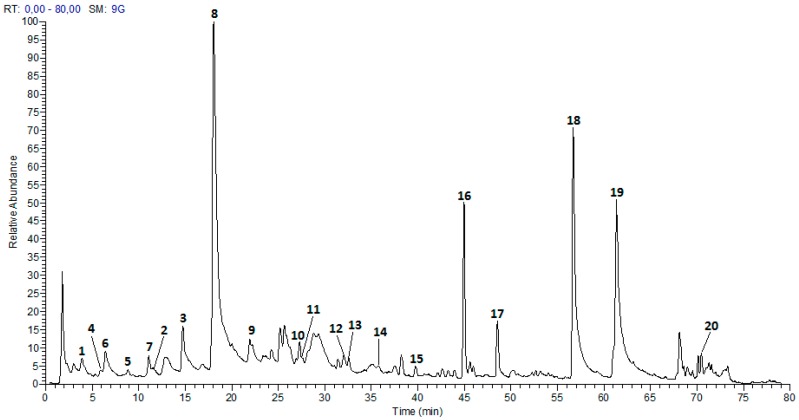
Chromatogram of *Phyllanthus acuminatus* enriched phenolic extract.

The extraction process, described in the Materials and Methods section, allowed us to obtain a phenolic enriched extract from *P. acuminatus* leaves. Table 1 summarizes the results of UPLC-ESI-MS analysis. Twenty compounds (Figure 1) were tentatively identified by comparing the fragmentation peaks with those reported in the literature, all of these compounds are reported for the first time in *P. acuminatus*, while previously reported in the genus *Phyllanthus*. As shown in Table 1, these compounds comprise eight flavonoids, including two flavanones—pinocembrin and six derivatives from apigenin, quercetin, and kaempferol; seven ellagitannins, two flavan-3-ols (prodelphinidin B dimer and (epi)gallocatechin); and three phenolic acids (ellagic acid, trimethylellagic acid, and ferulic acid).

**Figure 2:**
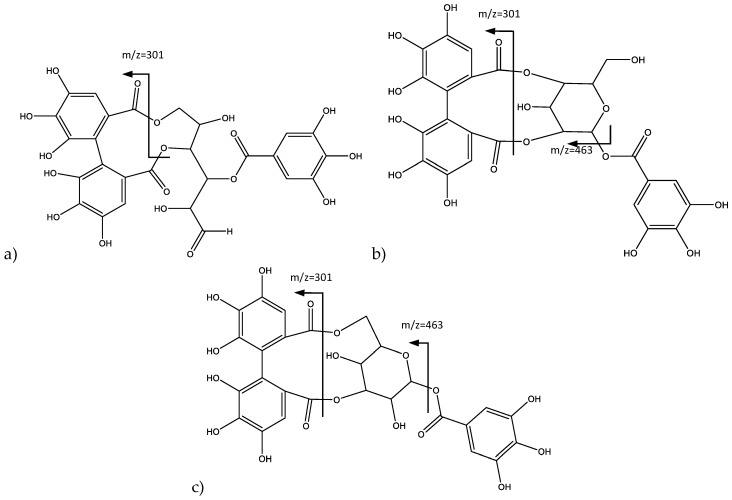
Structure and fragments of: (**a**) Gemin D (**1**); (**b**) Pinocembrin (**2**); and (**c**) Coraligin (**3**).

Peaks 1 (Rt = 3.89 min), 2 (Rt = 11.50 min), and 3 (Rt = 14.72 min) show negative molecular ion peaks [M − H]^−^ at *m*/*z* 633.0717, 633.0705, and 633.0701 (C_27_H_21_O_18_), respectively. The identified isomers are all part of the galloyl-HDDP-glucose type [20], corresponding tentatively to three isomeric ellagitannins, gemin D, phyllanemblinin B, and corilagin, respectively, all of them previously reported in the genus *Phyllanthus* [6]. In fact, for peak 3, as shown in Figure 2a, the 463 amu (atomic mass units) fragment was consistent with the loss of a galloyl acid, and the 301 amu fragment was consistent with the loss of the galloyl-glucose, which is coincident with the fragmentation previously reported for corilagin (**3**) [21,22].

On the other hand, Peak 2 also shows fragments at 463 amu and 301 amu, but additionally, an important peak is observed at 615 amu [M − H-H_2_O]. Thus, the loss of galloyl acid and galloyl-glucose, similar to corilagin, would correspond in turn to phyllanemblinin B (**2**) (Figure 2b). Peak 1 does not show the fragment at 463 amu attributed to the loss of galloyl bonded to a glucose, indicating the structure of gemin D (**1**) (Figure 2c), since this isomer does not have a glucose moiety and generates the loss of a fragment at 301 amu due to the structure bonded to the HHDP unit.

**Figure 3:**
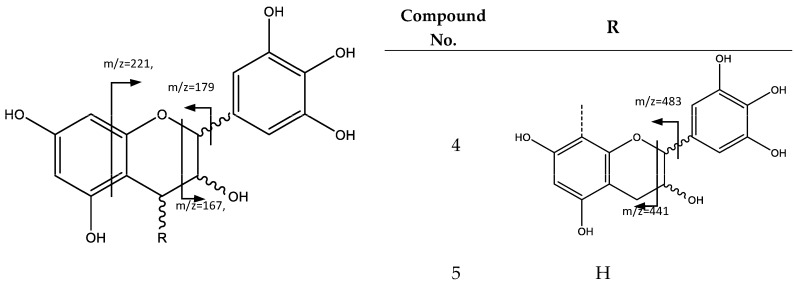
General structure and fragments of flavan-3-ols (epi)gallochatechin (**5**) and Prodelphinidin B dimer (**4**).

Peak 4 (Rt = 5.89 min) and peak 5 (Rt = 8.84 min) show negative molecular ion peaks [M − H]^−^ at *m*/*z* 609.1242 (C_30_H_25_O_14_) and *m*/*z* 305.0657 (C_15_H_13_O_7_), respectively, corresponding to flavan-3-ol structures. In fact, peak 5 presents a fragmentation pattern common for an (epi)gallocatechin monomeric unit (**5**), because of characteristic fragments at *m*/*z* 261, 221, 219, 179, 167, 165, consistent with the loss of CO_2_, C_4_H_4_O_2_, C_4_H_6_O_2_, C_6_H_6_O_3_, C_7_H_6_O_3_, and C_7_H_8_O_3_, respectively. The loss of C_4_H_4_O_2_ and C_4_H_6_O_2_ occurs because of the fragmentation of the flavan-3-ol A ring. C_6_H_6_O_3_ loss is due to the fission of the heterocyclic ring, while neutral fragments C_7_H_6_O_3_ and C_7_H_8_O_3_ are released through a retro-Diels–Alder fission [23]. In turn, peak 4 corresponds to prodelphinidin B dimer (**4**), since the MS2 spectrum shows an ion at *m*/*z* 483, which is the product of the heterocyclic ring fission, while *m*/*z* 441 belongs to the retro-Diels–Alder fission, and further loss of water originates the fragment observed at *m*/*z* 423 [23], as shown in Figure 3.

**Figure 4:**
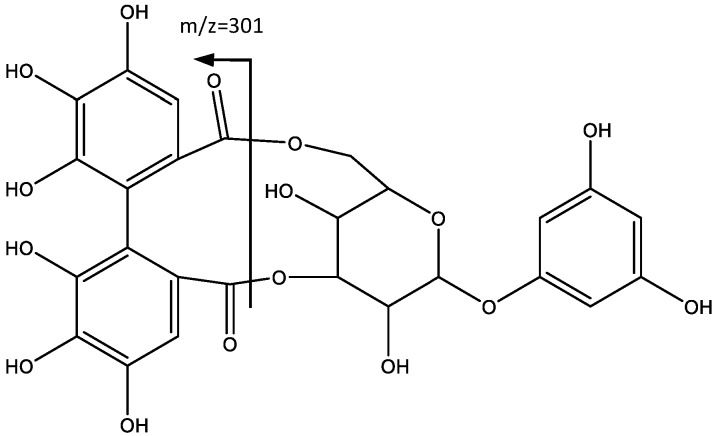
Structure and fragmentation of 1′,3′,5′-trihydroxybenzene 1′-*O*-[4,6-(*S*)-HHDP]-β-glucoside (**6**).

Peak 6 (Rt = 6.42 min) and peak 7 (Rt = 11.09 min) show negative molecular ion peaks [M − H]^−^ at *m*/*z* 589.0815 (C_26_H_21_O_16_) and *m*/*z* 913.1857 (C_45_H_37_O_21_) respectively, with a similar fragmentation pattern in agreement with an ellagitannin structure, including 1,3,5-trihydroxybenzene and HHDP moieties. For instance, peak 6 shows (MS2 spectrum) a fragment at *m*/*z* = 301 [M − H-288], which undergoes further fragmentation in a pattern that matches that of the HHDP group [24,25]. The loss of 288 amu is consistent with a hexose bonded to an 1,3,5-trihydroxybenzene, which leads to the identification of the compound as 1′,3′,5′-trihydroxybenzene 1′-*O*-[4,6-(*S*)-HHDP]-β-glucoside (**6**), as shown in Figure 4 [26].

**Figure 5:**
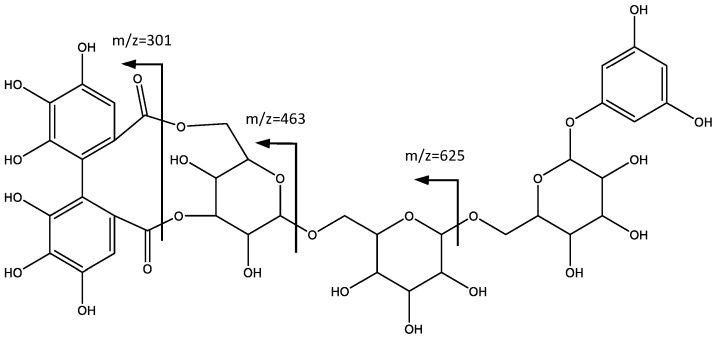
Structure and fragmentation of 11′,3′,5′-trihydroxybenzene 1′-*O*-[4,6-(*S*)-HHDP-β-glucosyl-β-glucosyl]-β-glucoside (**7**).

In a similar way, peak 7 shows fragments at *m*/*z* 301 that indicate the presence of the HHDP unit. As shown in Figure 5, the MS2 fragment at *m*/*z* 625 ([M − H]-288) is consistent with the loss of a glucose molecule bonded to a 1,3,5-trihydroxybenzene. Further losses of 162 amu at *m*/*z* 463 [M − H-288-162] and *m*/*z* 301 [M − H-288-162-162] are due to the loss of additional hexoses. Therefore, the compound is tentatively identified as 1′,3′,5′-trihydroxybenzene 1′-*O*-[4,6-(*S*)-HHDP-β-glucosyl-β-glucosyl]-β-glucoside (**7**).

**Figure 6:**
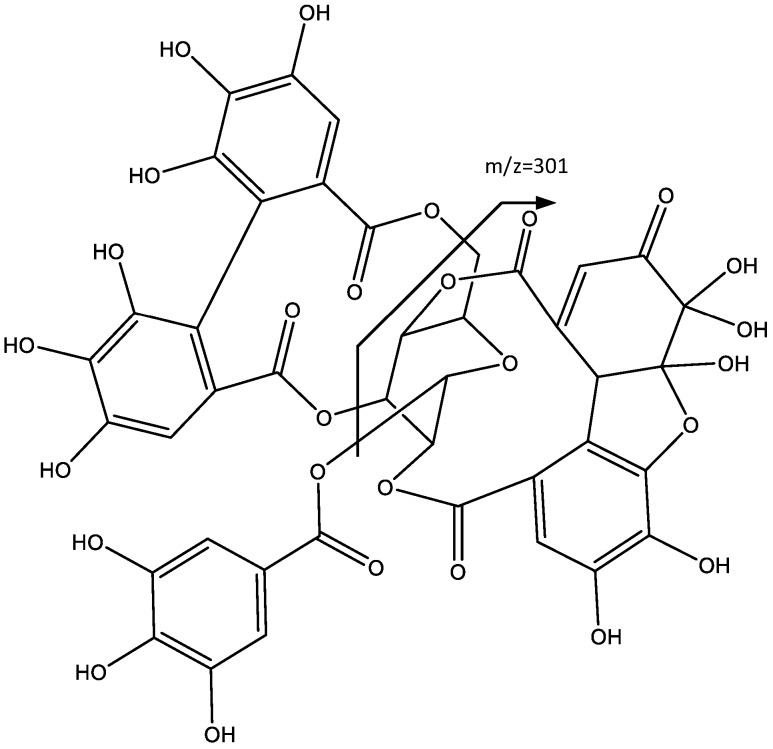
Structure and fragments of Geraniin (**8**).

Peak 8 (Rt = 18.03 min) shows a negative molecular ion peak [M − H]^−^ at *m*/*z* 951.0667, and it is tentatively assigned to Geraniin (**8**), ellagitannin with formula C_21_H_28_O_27_. In fact, as shown in Figure 6, the peak at *m*/*z* 933 corresponded to loss of H_2_O, and the fragments at *m*/*z* 915 and 897 match the successive loss of H_2_O, while the fragments at *m*/*z* 443 and 445 derive from the loss of galloyl and HDDP groups [27].

**Figure 7:**
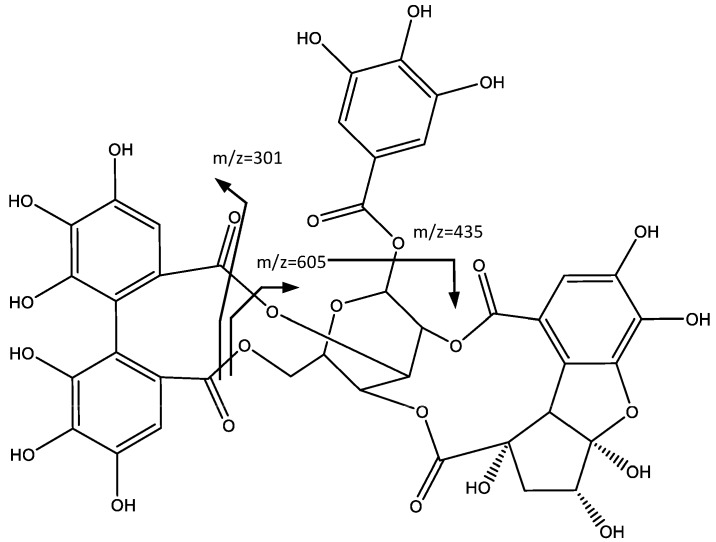
Structure and fragments of Phyllantusin C (**9**).

Peak 9 (Rt = 21.92 min) shows a negative molecular ion peak [M − H]^−^ at *m*/*z* 925.0905, and it is tentatively assigned to ellagitannin Phyllanthusiin C (**9**), whereas the peak at *m*/*z* 907 is due to the water loss in the geminal alcohol, which after further loss of HDDP, forms the fragment at *m*/*z* 605, as shown in Figure 7. The additional loss of a galloyl group originates the fragment at *m*/*z* 435. The peak at *m*/*z* 301 corresponds to the ionized HDDP unit [25].

**Figure 8:**
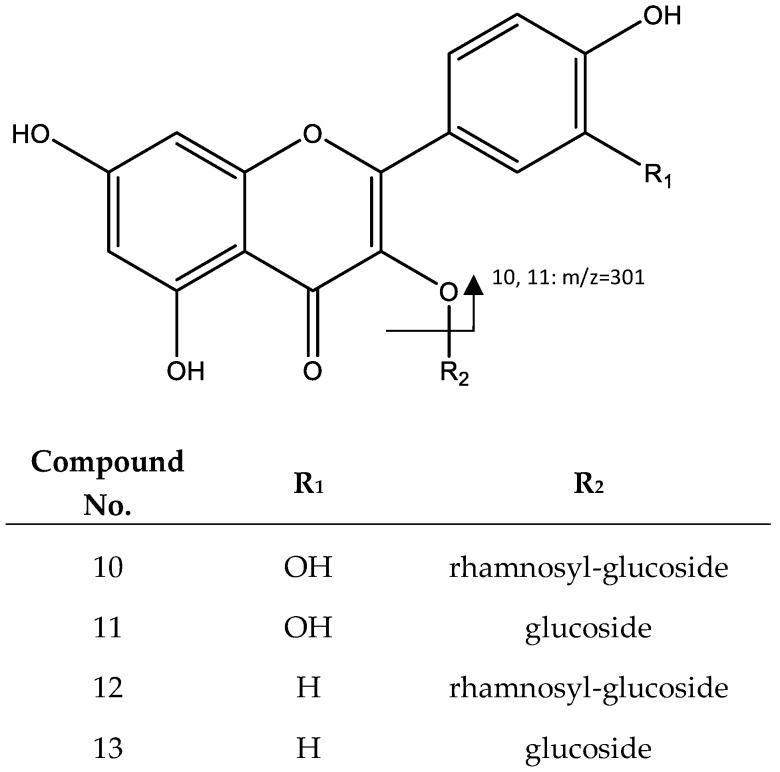
Structure and fragments of quercetin derivatives (**10**), (**11**), and kaempferol derivatives (**12**), (**13**).

Two flavonoids correspond to peak 10 (Rt = 27.28) and peak 11 (RT = 27.58), which show a negative molecular ion peak [M − H]^−^ at *m*/*z* 609.1430 and *m*/*z* 463.0857, respectively. Peak 10 was identified as quercetin-3-*O*-rutinoside (**10**), whose loss of the glycosylated unit yields the peak at *m*/*z* 301, followed by a fragmentation (Figure 8) with a pattern previously reported for the same compound [28], while peak 11 was assigned to quercetin-3-*O*-glucoside (**11**), because the main fragment corresponds to the loss of a hexose (*m*/*z* = 301, [M − H-162]) [25].

Peaks 12 and 13 were identified as kaempferol derivatives. For instance, Peak 12 (Rt = 31.95 min) shows a negative molecular ion peak [M − H]^−^ at *m*/*z* 593.1487, and it was identified as kaempferol-3-*O*-rutinoside (**12**) [29]; and peak 13 (Rt = 32.22 min), which shows a negative molecular ion peak [M − H]^−^ at *m*/*z* 447.0917, was identified as kaempferol-3-*O*-hexoside (**13**) [30]. Fragmentation for both molecules show the loss of glycosides generating signals that correspond to kaempferol (*m*/*z* 284 and 285), as shown in Figure 8.

**Figure 9:**
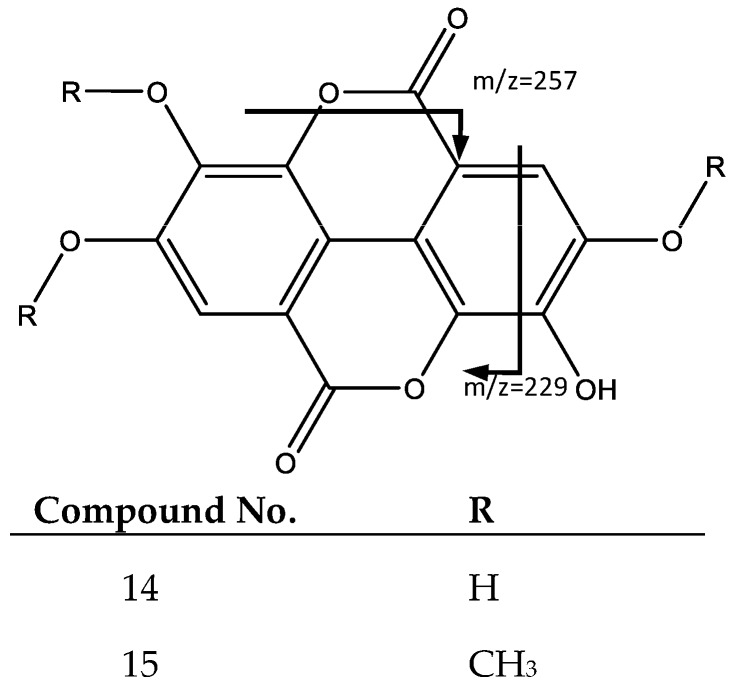
Structure and fragments of ellagic acid (**14**) and *O*-trimethyl ellagic acid (**15**).

Peaks 14 and 15 have similar fragmentation patterns in agreement with an ellagic acid moiety, as shown in Figure 9. For instance, peak 14 (Rt = 35.75 min) corresponds to ellagic acid (**14**), which shows a negative molecular ion peak [M − H]^−^ at *m*/*z* 300.9979, characterized by fragments at *m*/*z* 257 [M − H-CO_2_]^−^ and *m*/*z* 229 [31]. In turn, Peak 15 (Rt = 39.74 min) shows a negative molecular ion peak [M − H]^−^ at *m*/*z* 343.0443, and corresponds to *O*-trimethyl ellagic acid (**15**), whose fragments at *m*/*z* 328, 313, and 298 match the successive loss of methyl groups (–CH3) [32].

**Figure 10:**
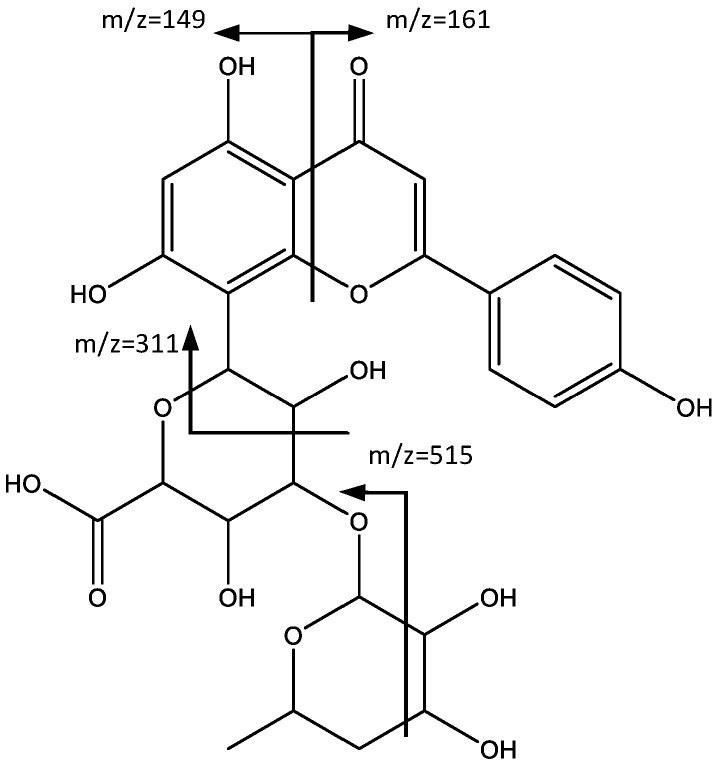
Structure and fragments of apigenin derivative (**16**).

Peaks 16 (Rt = 44.98 min) and 17 (Rt = 48.55 min) correspond also to flavonoids, specifically to apigenin and chrysin derivatives. In fact, peak 16 showed a negative molecular ion [M − H]^−^ at *m*/*z* 575.1381 (C_27_H_27_O_14_), and a fragment at *m*/*z* 515 corresponding to [M − H-60] which was previously reported [33] for this type of structure (Figure 10). According to Wu et al. [34], the fragment observed at *m*/*z* 311 corresponds to apigenin derivative (**16**). The fragment at *m*/*z* 293 arose from the successive loss of water. Two more peaks, observed at *m*/*z* 161 and *m*/*z* 149, originated from the fragment identified at *m*/*z* 311, due to the breakdown of the C ring, with each peak corresponding to one of the fragments generated.

**Figure 11:**
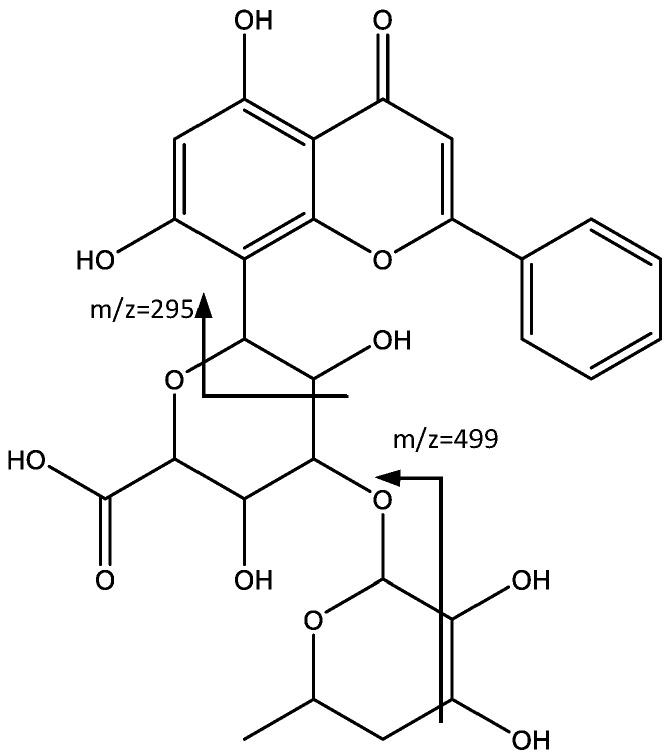
Structure and fragments of chrysin derivative (**17**).

In a similar way, peak 17 shows a negative molecular ion peak [M − H]^−^ at *m*/*z* 559.1428 (C_27_H_27_O_13_), which in turn shows a fragmentation pattern similar to the previous peak. For instance, the peak at *m*/*z* 499 originating from the loss of 60 amu [M − H-60] [33], and the peak at *m*/*z* 295, arises from a fragmentation similar to the one described by Wu et al. [34], with an oxygen in the aglycone instead of the glycoside, which leads to the tentative identification of chrysin (**17**) as aglycone (Figure 11).

**Figure 12:**
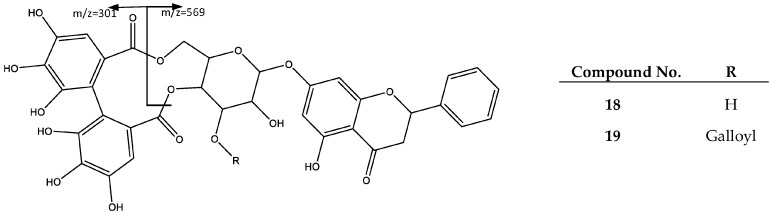
Structure and fragments of flavanone pinocembrin derivatives (**18**), (**19**).

Peaks 18 and 19 were assigned to ellagitannin derivatives of flavanone pinocembrin, previously reported in the genus *Phyllanthus* [6]. In fact, peak 18 (Rt = 56.67 min) shows a negative molecular ion peak [M − H]^−^ at *m*/*z* 719.1230 (C_35_H_27_O_17_), and was assigned to Pinocembrin 7-*O*-[4″,6″-(*S*)-hexahydroxydibenzoyl]-b-d-glucopiranoside (**18**), for which the main fragment at *m*/*z* = 301 corresponds to the HDDP group [35]. Peak 19 (Rt = 61.31 min) shows a negative molecular ion peak [M − H]^−^ = 719.1230 (C_42_H_31_O_21_) consistent with Pinocembrin 7-*O*-[3″-*O*-galloyl-4″,6″-(*S*)-hexahydroxydibenzoyl]-β-d-glucopiranoside (**19**), which besides the fragment at *m*/*z* 301 previously explained, exhibits fragments at *m*/*z* 569 due to HDDP loss, and at *m*/*z* 827 after the loss of a CO_2_ molecule, as shown in Figure 12 [25].

Finally, Peak 20 (Rt = 70.53 min) shows a negative molecular ion peak [M − H]^−^ at *m*/*z* 193.0490, and MS2 fragments at *m*/*z* 178 [M − H-H_2_O], 149 [M − H-CO_2_], and 134 [M − H-H_2_O-CO_2_], corresponding to ferulic acid [36].

### 2.2. Isolation and Characterization of Justicidin B

**Figure 13:**
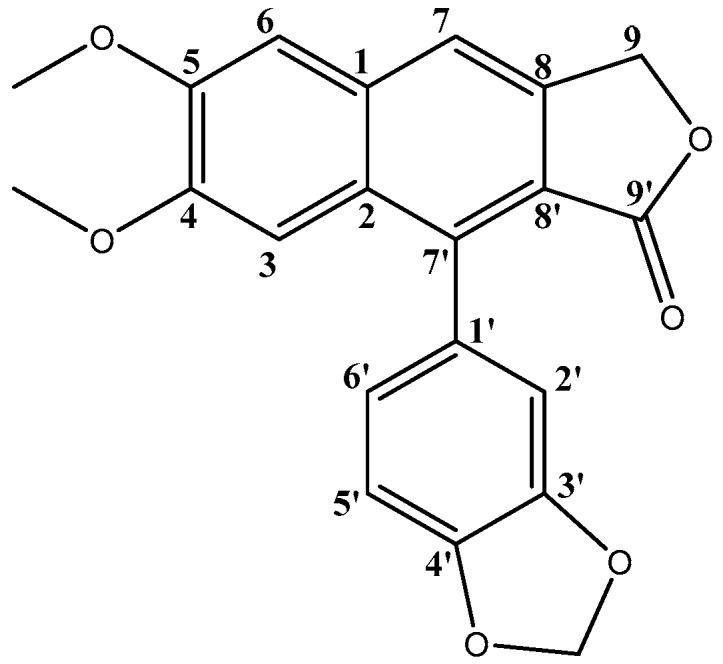
Justicidin B (**20**) structure.

As described in the Methods and Materials section, fractioning with Sephadex LH-20 of the non-polar extract of *P. acuminatus*, allowed the isolation of a compound characterized by ^1^H-NMR, ^13^C-NMR and 2D-(HMBC, HSQC, COSY)-NMR data coincident with reports from the literature [37,38,39] on aryl naphthalene lignan Justicidin B (**20**) C_21_H_16_O_6_ (Figure 13), a metabolite previously reported in *P. acuminatus* [7] and extensively studied because of its high antitumoral potential against different cancer cell lines [40], thus enabling a comparison of bioactivities among this metabolite and the phenolic extract, as described in the following sections.

### 2.3. Antioxidant Activity

DPPH is widely used as an indicator of antioxidant activity, although is not present naturally in the body, because it is a reliable assay that can give a preliminary appraisal of the antioxidant capacity of the agent under test [41]. As a second assay with better correlation to physiological radicals, our study used the ORAC method, which measures antioxidant scavenging activity against a peroxyl radical derived from 2,2-azobis(2-amidinopropane) dihydrochloride (AAPH), a hydrophilic alkyl peroxyl radical similar to the ones formed in biological systems, particularly in the process of lipid peroxidation [42].

Results obtained for antioxidant activity evaluation of both *P. acuminatus* extract and isolated Justicidin B metabolite, as well as ascorbic acid used as positive control, through DPPH and ORAC methodologies, are summarized in Table 2.

In both methodologies, *P. acuminatus* extract showed better results than Justicidin B and ascorbic acid, used as positive control. While no previous data has been reported for ORAC, the DPPH IC_50_ value of 14.28 µg/mL obtained for Justicidin B is in agreement with the moderate scavenging result previously reported in the literature in similar assay conditions [43]. Of special interest is the DPPH value for *P. acuminatus* extract (IC_50_ = 0.15 µg/mL), which is superior to DPPH values reported for *Phyllanthus* species in similar assay conditions, such as those reported for a phenolic-enriched extract of *P. niruri* (IC_50_ = 6.40 µg/mL) [44], and aqueous extracts of *P. emblica* (IC_50_ = 6.99–7.72 µg/mL) [45].

As described, the phenolic characterization of *P. acuminatus* extract indicated flavonoids and ellagitannins as main components, which have been previously reported as metabolites of interest for their antioxidant activity. For instance, flavones and ellagitannins have shown better antioxidant properties than other important phenolics, such as anthocyanins [46]; and, on the other hand, ellagitannins-enriched extracts exhibited greater inhibition than crude extracts from *R. idaeus* and *R. chamaemorus* [47].

### 2.4. Cytotoxicity Evaluation

**Figure 14:**
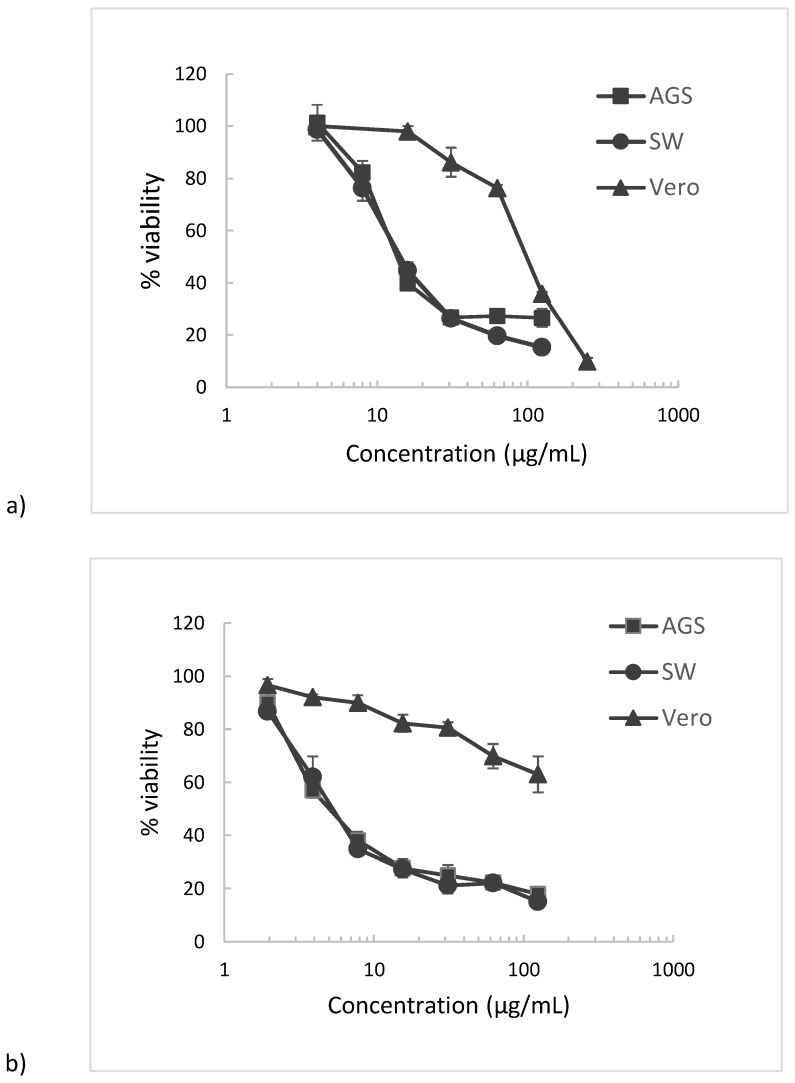
Cytotoxicity dose–response curves ^1^ of (**a**) Justicidin B and (**b**) *P. acuminatus* extract treatments on AGS and SW620 tumor cell lines and Vero normal cell lines. ^1^ Results are presented as mean ± SD of three independent experiments.

Table 3 summarizes the IC_50_ values for the cytotoxicity of *P. acuminatus* phenolic extract and Justicidin B on AGS human gastric adenocarcinoma, SW620 human colon adenocarcinoma, and Vero monkey normal epithelial kidney cell lines. IC_50_ values indicate that there is significant difference (one-way analysis of variance, ANOVA) between cytotoxicity values (*p* < 0.05) against gastric AGS adenocarcinoma cells and SW620 adenocarcinoma cells between *P. acuminatus* extract and Justicidin B metabolite, showing superior values for the plant extract. Also, Figure 14 shows dose-response curves.

In fact, the results obtained in the cytotoxic assay show that *P. acuminatus* extract exhibits a strong cytotoxic response in both AGS and SW620 cell lines (IC_50_ = 11.3 µg/mL and 10.5 µg/mL respectively). The isolated Justicidin B IC_50_ values indicate also good cytotoxicity (AGS: 19.5 µg/mL, SW: 24.8 µg/mL), however, the extract values are higher than those of Justicidin B for both in vitro cell phenotypes. Regarding normal Vero cells, ANOVA shows significant difference for both samples when comparing to cancer cells with lower cytotoxicity results, with the extract results indicating better values (IC_50_ = 226.6 µg/mL) compared to Justicidin B (IC_50_ = 104 µg/mL).

Regarding aryl naphthalene lignans, a similar study on Justicidin B using MTT assay with 72 h of incubation, showed moderate cytotoxicity on MDA-MB-231 and MCF-7 breast cancer cell lines (converted values of 38.91 and 14.09 μg/mL, respectively) [14] While no previous data has been reported for *P. acuminatus* extracts, the assessment of cytotoxicity has been performed for other species in the genus *Phyllanthus.* For instance, a study in similar MTT assay conditions performed on *P. niruri* phenolic extract indicated IC_50_ values of 113.2 µg/mL on the same cell line, AGS gastric, and 145.2 µg/mL on SW620 colon tumor cells [44], while on other adherent cell lines, MTT assay evaluation of hydro-methanolic extracts of *P. amarus* and *P. virgatus* on Hep G2 hepatic carcinoma, with measurements performed after 24 h of incubation, reported lack of cytotoxicity (IC_50_ > 250 µg/mL) [48].

Concerning the selectivity of the cytotoxic activity of samples between cancerous and normal cells, our results indicate significant differences (ANOVA, *p* < 0.05) between IC_50_ values for both samples in both AGS and SW620 adenocarcinoma cell lines, compared to IC_50_ values for normal Vero cells. When evaluating the selectivity index (SI), defined as the ratio of IC_50_ values of normal cells to cancer cells (AGS or SW620), *P. acuminatus* extract showed the highest selectivity result for SW620 colon cancer cells (SI = 21.5), followed closely for AGS gastric cancer cells (SI = 20.1), while Justicidin B results showed SI of 5.4 and 4.2 respectively, thus, lower selectivity values than *P. acuminatus* extract for both AGS and SW620 cancer cell lines. When comparing these selectivity results with reports using similar MTT assay conditions, Justicidin B displayed non-specific cytotoxicity in normal peripheral blood mononuclear cells (PMBC), HepG2 hepatoma, and PC3 prostrate tumor cell lines [40], while *P. niruri* phenolic extract showed selectivity values of 2.2 and 2.8, respectively, for AGS gastric and SW colon cancer cells [44]. Although selectivity (SI ≥ 3) and cytotoxicity (IC_50_ ≤ 20) results for *P. acuminatus* extract on AGS gastric and SW620 colon cancer cells fall within the parameters of the National Cancer Institute (NCI) [49,50] for plant extracts to be considered promising in the preliminary assay, further studies on mechanisms of action are needed.

The fact that polyphenols have control over several signaling pathways that affect different processes at cellular and tissue level, makes a synergic approach a conducive way to interpret the events mediated by polyphenolic profile. For instance, referring to the type of phenolic compounds present in the *P. acuminatus* extract, studies on quercetin and kaempferol flavonoid derivatives indicated that these compounds bioactivities—such as anti-oxidant, anti-inflammatory, and anti-proliferative—could act in a synergistic manner, and may repress carcinogenesis and cancer progression [51,52,53]. On the other hand, the flavone chrysin has been reported to induce apoptosis in several cancer cells [54], such as U87-MG malignant glioma [55], and U937 leukemia cells [56,57]. Also, flavone apigenin derivatives, such as vitexin, have been reported to induce apoptosis and inhibit autophagy on hepato-carcinoma cells (HCC) [58]. With respect to the other main group of polyphenolic compounds present in *P. acuminatus* extract, ellagitannins, such as pinocembrin derivatives, have shown to act on multiple molecular targets that are related to the inflammatory pathway in cancer cells [59]. In clinical studies, pomegranate juice rich in ellagitannins administered to patients with prostate cancer led to a decrease in the rate of rise of prostate specific antigen after primary treatment [60].

The diverse structure contents of *P. acuminatus* enriched phenolic extract could suggest the preliminary results on cytotoxicity and selectivity towards AGS gastric and SW620 colon cancer cells, are due to a multi-targeted, synergistic effect, however, further studies are needed to elucidate mechanisms of action.

## 3. Materials and Methods

### 3.1. Materials, Reagents, and Solvents

*Phyllanthus acuminatus* leaf plant material was acquired from a local Agricultural Producers Association in the Caribbean region of Costa Rica. The plant was identified with the support of the Costa Rican National Herbarium, and a voucher is deposited there. *P. acuminatus* material was rinsed in water and cut into small pieces. Subsequently, it was dried in a stove at 40 °C until completely dry, and after being ground, it was preserved at −5 °C. Among reagents, 2,2′-azobis(2-methyl-propionamidine)-dihydrochloride (AAPH), fluorescein, ascorbic acid, 3-(4,5-dimethylthiazol-2-yl)-2,5-diphenyltetrazolium bromide (MTT), trypsin-EDTA solution and Sephadex LH-20 gel were provided by Sigma-Aldrich (St. Louis, MO, USA), while amphotericin B, penicillin–streptomycin, and Eagle’s minimum essential medium (MEM, 10% fetal bovine serum), were purchased from Life Technologies (Carlsbad, CA, USA). AGS human gastric adenocarcinoma, SW 620 human colorectal adenocarcinoma and Vero monkey normal epithelial kidney cell lines were obtained from American Type Culture Collection (ATCC, Rockville, MD, USA). DMSO was provided by Sigma-Aldrich (St. Louis, MO, USA), while MTBE, dichloromethane, chloroform, and methanol were purchased from Baker (Center Valley, PA, USA).

### 3.2. Extraction of P. acuminatus Secondary Metabolites

The process followed for obtaining a phenolic-enriched extract from *P. acuminatus* was formerly described on other plants by our group [61], involving different organic solvents to optimize separation of compounds in a preliminary effort for characterization. Briefly, plant dried material was first extracted in a mixture of methyl *tert*-butyl ether (MTBE) and methanol (MeOH) 90:10 (*v*/*v*) at 25 °C during 30 min in ultrasound. Afterwards, it was extracted for 24 h in order to obtain a non-polar extract of the samples. After filtration, the extraction was repeated once. The extracts were combined, and the solvents evaporated in a rotavapor to dryness, and subsequently washed with MeOH in order to extract any polyphenols. The residual plant material was extracted with MeOH at 25 °C during 30 min in ultrasound, and then extracted for 24 h. After filtration, the extraction was repeated twice. The three methanol extracts were combined with the previous MeOH washings, and were evaporated in a rotavapor to dryness. Finally, the dried extract was washed with hexane, MTBE and chloroform consecutively in order to obtain a phenolic rich extract. 

On the other hand, the non-polar extract after the MeOH washings, was concentrated and dissolved in CH_2_Cl_2_/MeOH 70:30 (*v*/*v*) and extracted twice with water 50:50 (*v*/*v*). The aqueous phase was extracted twice with CH_2_Cl_2_ 50:50 (*v*/*v*), and the organic phase was evaporated in a rotavapor to dryness. The extract was fractionated using Sephadex LH-20, allowing isolation of 24 mg (0.01%) of lignan 20, with the following NMR data. ^1^H-NMR (CDCl_3_) δ (ppm) 3.82 (s, 3H), 4.05 (s, 3H), 5.38 (d, *J* = 1.0 Hz, 2H), 6.06 (d, *J* = 1.5 Hz, 1H), 6.10 (d, *J* = 1.5 Hz, 1H), 6.83 (dd, *J* = 7.9, 1.7 Hz, 1H), 6.86 (d, *J* = 1.7 Hz, 1H), 6.97 (d, *J* = 7.9 Hz, 1H), 7.11 (s, 1H), 7.19 (s, 1H), 7.70 (s, 1H); and ^13^C-NMR (CDCl_3_) δ (ppm) 55.99 (MeO-C5), 56.22 (MeO-C4), 68.19 (C9), 101.40 (O–CH_2_–O), 106.01 (C6), 106.16 (C3), 108.38 (C5′), 110.73 (C6′), 118.42 (C7), 123.63 (C2′), 128.56 (C1′), 129.02 (C1), 133.33 (C2), 139.68 (C8′), 139.82 (C8), 147.70 (C4′), 147.74 (C3′), 150.23 (C5), 151.98 (C4), 170.12 C9′).

### 3.3. UPLC-ESI—MS Analysis

The UPLC-MS system used to analyze the phenolic composition of the *P. acuminatus* extract consisted of an LTQ Orbitrap XL mass spectrometer with an Accela 1250 binary Pump, a PAL HTC Accela TMO autosampler, a PDA detector (ThermoFisher Scientific, San Jose, CA, USA), and a G1316A column compartment (Agilent, Palo Alto, CA, USA). Separation was carried out on a Hypersil Gold AQ RP—C18 UHPLC column (200 mm × 2.1 mm i.d., 1.9 µm, Thermo Fisher Scientific, Waltham, Massachusetts) with an UltraShield pre-column filter (Analytical Scientific Instruments, Richmond, CA, USA) at a flow rate of 0.3 mL/min. Mobile phases A and B consist of a combination of 0.1% formic acid in water (*v*/*v*), and 0.1% formic acid in acetonitrile (*v*/*v*), respectively. The linear gradient is from 4% to 20% B (*v*/*v*) at 40 min, to 35% B at 70 min and to 100% B at 71 min, and held at 100% B to 75 min. The UV–vis spectra were acquired from 200–700 nm.

Negative electrospray ionization mode was used, and the conditions were set as follows: sheath gas, 70 (arbitrary units); aux and sweep gas, 15 (arbitrary units); spray voltage, 4.8 kV; capillary temperature, 300 °C; capillary voltage, 15 V; tube lens, 70 V. The mass range was from 100 to 2000 amu with a resolution of 30,000, FTMS AGC target at 2 × 10^5^, FT-MS/MS AGC target at 1 × 10^5^, isolation width of 1.5 amu, and max ion injection time of 500 ms. The most intense ion was selected for the data-dependent scan to offer their MS^2^ to MS^5^ product ions, respectively, with a normalization collision energy at 35%.

### 3.4. DPPH Radical-Scavenging Activity

A solution of 2,2-diphenyl-1-picrylhidrazyl (DPPH) (0.25 mM) was prepared using methanol as solvent. Next, 0.5 mL of this solution were mixed with 1 mL of sample or ascorbic acid used as positive control, at different concentrations, and incubated at 25 °C in the dark for 30 min. DPPH absorbance was measured at 517 nm. Blanks were prepared with 1 mL of each sample concentration and 0.5 mL of methanol instead of DPPH. The percentage of the radical-scavenging activity of the sample was plotted against its concentration to calculate IC_50_, which is the amount of sample required to reach the 50% radical-scavenging activity. The samples were analyzed in three independent assays each one in triplicate.

### 3.5. ORAC Antioxidant Activity

The ORAC (Oxygen Radical Absorbance Capacity) antioxidant activity was determined following a method previously described [62] using fluorescein as a fluorescence probe. Briefly, 0.05 g of each sample to be measured is mixed with 10 mL of methanol/HCl (1000:1, *v*/*v*) and, if needed, sonicated until complete dissolution, for 5 min. Afterwards, the mixture was centrifuged and filtered. The reaction was carried out in 75 mM phosphate buffer (pH 7.4) at 37 °C. The final assay mixture contained AAPH (12 mM), fluorescein (70 nM), and 20 µL of either Trolox (1–8 µM) or sample (extract or Justicidin B) at different concentrations. Blanks were prepared adding 20 µL of phosphate buffer instead of sample, and positive controls were prepared adding 20 µL of ascorbic acid at different concentrations, instead of sample. Fluorescence was recorded every minute for 98 min in black 96-well untreated microplates (Nunc, Denmark), using a Polarstar Galaxy plate reader (BMG Labtechnologies GmbH, Offenburg, Germany) with 485-P excitation and 520-P emission filters. Fluostar Galaxy software version 4.11-0 (BMG Labtechnologies GmbH, Offenburg, Germany) was used to measure fluorescence. Fluorescein was diluted from a stock solution (1.17 mM) in 75 mM phosphate buffer (pH 7.4), while AAPH and Trolox solutions were freshly prepared. Samples were evaluated in three independent experiments with different concentrations of each sample (or positive control) analyzed in triplicate.

Fluorescence values obtained were normalized to the curve of the blank (no antioxidant). The area under the fluorescence decay curve (AUC) was calculated from the normalized curves, and the net AUC was then established. Subsequently, regression equation between antioxidant concentration and net AUC was obtained. Finally, ORAC value was estimated by dividing the slope of the latter equation by the slope of the Trolox line. ORAC values were expressed as mmol of Trolox equivalents (TE)/g of extract.

### 3.6. Evaluation of Cytotoxicity

The AGS, SW620, and Vero cells were grown in MEM (10% FBS) in the presence of glutamine (2 mmol/L), penicillin (100 IU/mL), streptomycin (100 µg/L), and amphotericin B (0.25 µg/m), at 37 °C, in a humidified atmosphere (5% CO_2_). Briefly, 100 µL of 1.5 × 10^5^ cells/mL (suspension) were seeded overnight into 96-well plates to reach 100% confluency. Subsequently, the cells were exposed for 48 h to 50 µL of samples in concentrations varying 1.5–500 µg/mL in MEM (DMSO 0.1% *v*/*v*). Controls to establish 100% of viability were prepared, adding 50 µL of MEM (DMSO 0.1% *v*/*v*) instead of samples. Afterwards, the medium was eliminated, cells were washed with PBS (100 µL) and incubated with 100 µL of a MTT solution (0.5 mg/mL, final concentration) in PBS, for 2 h at 37 °C. Then, MTT was removed, and the formazan crystals were dissolved in 100 µL of ethanol 95%. Absorbance was read at 570 nm in a microplate reader. DMSO was diluted in media in the same way as the extracts and incubated with the cells for 48 h to be used as control. Dose–response curves were established, and IC_50_ was calculated. Samples were tested in three independent experiments with different doses of each sample analyzed in triplicate.

## 4. Conclusions

This study represents the first detailed MS analysis of phenolic-enriched extract from *P. acuminatus.* Using UPLC-ESI-MS techniques, 20 phenolic compounds were identified, comprising eight flavonoids, (two flavanones—pinocembrin isomers and six derivatives from apigenin, chrysin, quercetin and kaempferol); seven ellagitannins, two flavan-3-ols (prodelphinidin B dimer and (epi)gallocatechin); and three phenolic acids (ellagic acid, trimethylellagic acid and ferulic acid). These findings constitute the first report on the diversity of phenolics in *P. acuminatus.* DPPH and ORAC antioxidant methods were evaluated both in the extract and the isolated aryl naphthalene lignan Justicidin B, with *P. acuminatus* extract showing a particularly high value (IC_50_ = 0.15 µg/mL). Based on these results, due to antioxidant properties of flavonoids resulting in antipathogenic effects that can be non-specific [18], further studies on these properties could be promising. *P. acuminatus* phenolic-enriched extract also showed cytotoxicity and selectivity on AGS gastric and SW620 colon adenocarcinoma cell lines with SI > 20 for both cell lines when compared to normal cells, with lower values (SI > 4) for Justicidin B. Since polyphenols could work in a synergistic manner, purification or fractioning of *P. acuminatus* phenolic extract could be of interest to further evaluate the structure–bioactivity relationship. Also, studies using solvents adequate for human health consumption, such as ethanolic or aqueous phenolic-enriched extracts, are important to assess the potential anticancer bioactivity of *P. acuminatus* phenolic extracts and components. The results on the cell lines studied could suggest potential health effects of *P. acuminatus* extract on gut-related diseases, considering that polyphenols are metabolized by the gut [19,63]. However, further research is required to understand the mechanisms of action and pathways.

## Figures and Tables

**Table 1:** Phenolic characterization of *P. acuminatus* extract.

No.	Tenative Identification	t_R_ (min)	λ_max_ (nm)	[M − H]^+^	Formula	Error (ppm)	MS^2^
1	Gemin D	3.89	216, 265	633.0717	C_27_H_21_O_18_	1.738	[633]: 275(18), 301(100), 249(15)
2	Phyllanemblinin B	11.50	216, 278	633.0705	C_27_H_21_O_18_	3.633	[633]:463(26), 301(100), 275(15), 614(62), 615(24)
3	Corilagin	14.72	221, 269	633.0701	C_27_H_21_O_18_	4.265	[633]: 463(27), 301(100), 275(15)
4	Prodelphinidin B dimer	5.89	205, 270	609.1242	C_30_H_25_O_14_	−0.328	[609]:305(50), 423(85), 441(100), 483(28), 591(18)
5	(epi)galocatequina	8.84	205, 270	305.0657	C_15_H_13_O_7_	1.311	[305]: 125(24), 165(30), 219(77), 179(100), 261(41), 287(12), 247(13), 221(84), 167(10)
6	1′,3′,5′-Trihydroxybenzene 1′-*O*-[4,6-(*S*)-HHDP]-β-Glucoside	6.42	199, 271	589.0815	C_26_H_21_O_16_	−1.478	[589]: 301(100)
7	1′,3′,5′-Trihydroxybenzene 1′-*O*-[4,6-(*S*)-HHDP-β-Glucosyl-β-Glucosyl]-β-Glucoside	11.09	204, 264	913.1857	C_45_H_37_O_21_	−3.285	[913]: 625(100), 463(13)
8	Geraniin	18.03	230, 276	951.0667	C_41_H_27_O_27_	−4.676	[951]: 933(100)
9	Phyllanthusiin C	21.92	222, 278	925.0905	C_40_H_29_O_26_	−4.540	[925]: 301(100), 435(15)605(10)907(13)
10	quercertin-3-*O*-rutinósido	27.28	219, 255, 349	609.1430	C_27_H_29_O_16_	4.268	[609]:343(8), 301(100), 300(39)
11	quercetin-3-*O*-hexoside	27.58	221, 254, 347	463.0857	C_21_H_19_O_12_	4.319	[463]: 301(100), 300(35)
12	kaempferol-3-*O*-rutinoside	31.95	221, 271	593.1487	C_27_H_29_O_15_	−2.866	[593]: 285(100)
13	kaempferol-3-*O*-hexoside	32.22	221, 265	447.0917	C_21_H_19_O_11_	2.237	[247]: 285(69), 284(100), 255(17), 327(18)
14	Ellagic acid	35.75	221, 265	300.9979	C_14_H_5_O_8_	1.661	[301]: 257(100), 229(60), 301(28), 284(23), 185(28), 255(12), 201(11)
15	*O*-trimethyl ellagic acid	39.74	222, 243, 353, 366	343.0443	C_17_H_11_O_8_	−3.207	[343]: 328(100)
16	Apigenin derivative	44.98	199, 227, 287	575.1381	C_27_H_27_O_14_	3.477	[575]: 515(80), 455(16), 371(11), 343(10), 311(100)
17	Chrysin derivative	48.55	223, 289	559.1428	C_27_H_27_O_13_	4.292	[559]:499(100), 295(57)
18	Pinocembrin 7-*O*-[4″,6″-(*S*)-hexahydroxydibenzoyl]-b-d-glucopiranoside	56.67	226, 282	719.1230	C_35_H_27_O_17_	−2.503	[719]: 301(100)
19	Pinocembrin 7-*O*-[3″-*O*-galloyl-4″,6″-(*S*)-hexahydroxydibenzoyl]-β-d-glucopiranoside	61.31	223, 282	871.1323	C_42_H_31_O_21_	−4.018	[871]: 301(100), 569(13), 827(13)
20	Ferulic acid	70.53	224	193.0490	C_10_H_9_O_4_	−3.698	[193]: 178(70), 149(100), 134(62)

**Table 2:** Antioxidant activity of *P. acuminatus* phenolic extract and Justicidin B metabolite.

Sample	DPPH ^1,2^ IC_50_ (μg/mL) ± SD	ORAC ^1,2^ (mmol TE/mg Extract) ± SD
*P. acuminatus* extract	0.15 ^a^ ± 0.01	2.76 ^a^ ± 0.05
Justicidin B	14.28 ^b^ ± 0.30	0.95 ^b^ ± 0.02
Ascorbic Acid	3.74 ^c^ ± 0.05	1.62 ^c^ ± 0.07

^1^ Different superscript letters in the same column indicate differences are significant at *p* < 0.05 using ANOVA with a Tukey post hoc test; ^2^ Results represent average ± standard deviation from three independent runs for each sample (*n* = 3).

**Table 3:** Cytotoxicity of *P. acuminatus* extract and Justicidin B to gastric (AGS) and colon (SW620) adenocarcinoma cells as well as to control Vero cells.

Sample	IC_50_ (µg/mL) ± S.D. ^1^		
	AGS ^2^	SW620 ^2^	Vero ^2^
*P. acuminatus* extract ^3^	11.3 ^a,^* ± 0.7 (SI = 5.4)	10.5 ^a,^* ± 0.5 (SI = 20.1)	226.6 ^a,^^◊^ ± 4.2
*Justicidin B* ^3^	19.5 ^b,^* ± 2.2 (SI = 4.2)	24.8 ^b,^* ± 2.1 (SI = 21.5)	104 ^b,^^◊^ ± 6

^1^ Results are presented as mean ± SD of three independent experiments. ^2^ Different superscript letters in the same column indicate differences are significant at *p* < 0.05 using ANOVA with a Tukey post hoc test. ^3^ Different superscript signs in the same row indicate differences are significant at *p* < 0.05 using ANOVA with a Tukey post hoc test.
